# Airway specific deregulation of asthma-related serpins impairs tracheal architecture and oxygenation in *D. melanogaster*

**DOI:** 10.1038/s41598-024-66752-0

**Published:** 2024-07-17

**Authors:** Birte Ehrhardt, Hanna Angstmann, Beate Höschler, Draginja Kovacevic, Barbara Hammer, Thomas Roeder, Klaus F. Rabe, Christina Wagner, Karin Uliczka, Susanne Krauss-Etschmann

**Affiliations:** 1 Division of Early Life Origins of Chronic Lung Diseases, Research Center Borstel, Airway Research Center North (ARCN), German Center for Lung Research (DZL), Parkallee 1, 23845 Borstel, Germany; 2 DZL Laboratory for Experimental Microbiome Research, Research Center Borstel, Airway Research Center North (ARCN), German Center for Lung Research (DZL), Borstel, Germany; 3 Division of Molecular Physiology, Institute of Zoology, Christian-Albrechts University Kiel, Kiel, Airway Research Center North (ARCN), German Center for Lung Research (DZL), Borstel, Germany; 4 Department of Pneumology, LungenClinic, Grosshansdorf, Germany; 5 Department of Medicine, Christian Albrechts University, Kiel, Germany; 6 Division of Invertebrate Models, Priority Research Area Asthma and Allergy, Research Center Borstel, Borstel, Germany; 7 Institute of Experimental Medicine, Christian-Albrechts-University Kiel, Kiel, Germany

**Keywords:** Serpins, Airway structure, Development, Life-span, Oxygenation, *Drosophila melanogaster*, Genetics, Physiology, Experimental organisms, Morphogenesis

## Abstract

Serine proteases are important regulators of airway epithelial homeostasis. Altered serum or cellular levels of two serpins, *Scca1* and *Spink5*, have been described for airway diseases but their function beyond antiproteolytic activity is insufficiently understood. To close this gap, we generated fly lines with overexpression or knockdown for each gene in the airways. Overexpression of both fly homologues of *Scca1* and *Spink5* induced the growth of additional airway branches, with more variable results for the respective knockdowns. Dysregulation of *Scca1* resulted in a general delay in fruit fly development, with increases in larval and pupal mortality following overexpression of this gene. In addition, the morphological changes in the airways were concomitant with lower tolerance to hypoxia. In conclusion, the observed structural changes of the airways evidently had a strong impact on the airway function in our model as they manifested in a lower physical fitness of the animals. We assume that this is due to insufficient tissue oxygenation. Future work will be directed at the identification of key molecular regulators following the airway-specific dysregulation of *Scca1* and *Spink5* expression.

## Introduction

The airway epithelium represents the first barrier of protection against harmful airborne substances such as allergens, microbes and pollutants. By transmitting signals to underlying cells, the epithelium helps instruct innate and adaptive immune cells to respond specifically to distinct environmental challenges and is therefore essential for maintaining lung homeostasis^1^. Consequently, impaired epithelial functioning has been proposed to contribute to the pathogenesis of airway diseases^2,3^.

Proteases are key elements of the airway epithelial host defence and are tightly regulated by anti-proteases to prevent lung damage from an overshooting proteolytic activity. In line, an enhanced proteolytic activity has been described for a majority of chronic lung diseases such as asthma^4–7^, chronic obstructive pulmonary disease (COPD)^8,9^, cystic fibrosis (CF)^10,11^, non-CF bronchiectasis^12,13^ and primary ciliary dyskinesia^14–16^. Among anti-proteases, serpins constitute the largest family and are found in almost all organisms^17^. They act by neutralizing target proteases through irreversible binding upon which the respective serpin is also inactivated^18^. Two of the nine human clades, serpins A and B, have been associated with chronic lung diseases^19^. Group A serpins act extracellularly, with the absence of Alpha-1 antitrypsin being the best-known cause of alveolar destruction leading to emphysema^20^. Serpins of clade B function intracellularly where *SERPINB3*, also termed squamous cell carcinoma antigen-1 (*Scca1*), is an evolutionary conserved protease inhibitor expressed in the lung^21,22^. Contrary to its name, it inhibits the cysteine proteases cathepsin K, L, S and papain. Significantly higher serum levels of *Scca1* have been described in asthmatic children and adolescents as compared to healthy controls^23,24^ as well as in bronchial biopsies from patients with asthma^24^. In line, stimulation of primary human airway epithelial cells with the type 2 cytokines IL-4 and IL-13 increased the expression of *Scca1* and its close homologue *Scca2* (*SERPINB4*)^24,25^. In vitro-polarized T_H_2 cells from patients with grass pollen allergy expressed higher levels of both *SERPINB3* and *SERPINB4* mRNA compared with unpolarized CD4 T cells. CD27^−^CD4^+^ ILC2 from patients expressed higher levels of both *SERPINB3* and *SERPINB4* mRNA compared with ILC1. Knockdown of either *SERPINB3* or *SERPINB4* mRNA resulted in decreased viability of CD27-CD4 + compared with control transduced cells^26^. Further, increased *Scca1* levels were observed in epithelial lining fluid from COPD patients directly after smoking^27^.

*Spink5* (serine protease inhibitor Kazal-type 5) encodes the serine protease inhibitor LEKTI (Lympho-epithelial Kazal-type-related inhibitor) and was originally described for the Netherton syndrome^28^ which is a rare autosomal recessively inherited disease caused by *Spink5* mutations^29^. Symptoms of this syndrome include hair abnormalities, severe skin inflammation and high serum-IgE along with allergies prompting investigation in asthma. Polymorphisms within the *Spink5* gene have been linked to childhood but not adult asthma^30–33^. Rhinoviral (HRV) infection has been associated with asthma development^34–36^. Inoculation with HRV-16 upregulated *Spink5* in nasal samples from healthy individuals which was not seen in asthma patients^37^. Vice versa, *Spink5* was further found to be expressed at lower levels in the small airways in a mouse model of emphysema, and LEKTI was reduced in smokers with emphysema^38^, suggesting that LEKTI might be a key factor protecting from alveolar destruction. Although *Scca1* and *Spink5* domains have been implicated in airway diseases, the function of both serpins in the respiratory epithelium and consequently the host´s ability to cope with environmental stressors is still unclear. To address these knowledge gaps, the fruit fly *Drosophila melanogaster* is an apt model organism because its respiratory system is built exclusively of epithelial cells^39^. We therefore generated fly lines with airway-specific dysregulation of evolutionarily conserved gene orthologues of *Scaa1* and *Spink5* to investigate firstly their effects on airway morphology and physiology and secondly the resulting ability of flies to cope with an environmental stressor.

## Material and methods

### Selection of orthologous genes

Candidates of orthologous genes for *Scca1* and *Spink5* were identified (27 genes for *SERPINB3* and 17 genes for *Spink5*) using flybase.org^40^. Candidates without inhibitor-region or kazal-domain were excluded. The homology of amino acid sequences of the remaining candidate genes was identified by NCBI BLAST^41^. Only candidates with a sequence identity of > 30% were chosen, yielding 10 candidate genes for *SERPINB3* and 5 candidate genes for *Spink5.* The expression level of these 15 genes were quantified in wild-type Canton S flies in whole larvae and isolated trachea by qRT-PCR (Fig. S1). Finally, one candidate gene with the highest expression level in the tracheae was chosen for further experiments. This way the fly gene *Spn43Aa* was identified as orthologous gene for *Scca1* and *CG14933* as an orthologue for *Spink5*.

### Isolation of tracheae, RNA isolation and qRT-PCR

To quantify gene expression in the airways, tracheae were isolated from L3 larvae on day 5 after oviposition as described earlier^42^. RNA isolation from the tracheae was performed following the protocol of the NucleoSpin RNA II Kit (Macherey–Nagel). Total-RNA was eluted in 40 µl RNAse-free water and due to low concentration no extra DNA digestion was done. Concentration of total-RNA was measured at the nanophotometer Implen (P330) using a factor of 10. cDNA was transcribed from the total-RNA using SuperScript^©^ III reverse transcriptase according to the protocol of the manufacturer. For each transcription 250 ng total-RNA was used in a total reaction volume of 20 µl. To quantify gene expression, cDNA was quantified using qRT-PCR. For this purpose, cDNA was first diluted 1:1 with RNAse free water and mixed with LightCycler^©^480 SYBR Green I Master as well as sense and antisense Primer. qRT-PCR program was run in 45 amplification cycles (Table S2). The sequences of the primer are shown in Table S3. The expression level was calculated relative to the housekeeping gene *rpl32*.

### Breeding, husbandry and crossing of fly lines

All experiments were performed in *Drosophila melanogaster*. Flies were kept on standard medium at 25 °C and 50–60% humidity in a 12 h/12 h light/dark cycle. Overexpression and knockdown of the candidate genes in the larval tracheae was achieved by using the UAS-GAL4 system^43^. To drive expression specifically in the tracheae, the *ppk4*-Gal4 driver was chosen as *ppk4* is expressed in all epithelial compartments of the larval tracheal system^44^. It is therefore a suitable driver line for the investigation of morphological changes in the main conducting airways. For knockdown experiments we used the corresponding UAS-RNAi lines and for overexpression experiments, we used UAS lines allowing expression of the corresponding genes, targeted to the tracheal system by the *ppk4*-Gal4 driver. In detail, to obtain the knockdown of the genes in the tracheae *ppk4*-Gal4 was driving UAS-*CG14933*-RNAi or UAS-*spn43Aa*-RNAi. Respectively, overexpression was induced by ppk4-Gal4 driving UAS-*CG14933* or UAS-*spn43Aa*.

One week old virgin females of the UAS-effector-line and one week old males of the GAL4-driver-line were crossed for synchronized egg deposition and larvae of the same age were collected. UAS-RNAi fly lines were obtained from Bloomington Stock Center; Indiana. UAS-ORF fly lines were ordered from FlyORF^45^. For detailed information on the genotypes see Table S1.

### Assessment of tracheal morphology

The number and length of secondary branches of L1 and L3 larvae were measured by a modified protocol of Jones and Metzstein^46^. Heat-fixated larvae were analyzed at 40-fold (L1 larvae) or 20-fold (L3 larvae) magnification (BX51 microscope with DP25 camera, Olympus Deutschland GmbH, Hamburg). The third thoracic segment was captured and analyzed in ImageJ (version 2.1.4.7)^47^. Using the Plugin NeuronJ^48^ secondary branches were marked and measured.

### Fitness parameter

To characterize the fly lines and measure effects of exposures, three fitness parameters were monitored, namely survival rate and developmental time^49^ of larvae and pupae as well as lifespan of adults^50^.

The survival of developmental stages was calculated by counting formed pupae from a known number of larvae and counting eclosed adults from the known number of pupae. Subsequently data was calculated and is shown as percent of larvae used.

The developmental time of larvae (referred to as larval developmental time) was assessed at defined timepoints^49^ and the mean developmental time of larvae was defined as the timepoint when 50% of the larvae had gone into pupation. Accordingly, the development of pupae (referred to as metamorphosis time) was assessed at defined timepoints and the mean developmental time of pupae was defined as timepoint when 50% of adults eclosed. It was calculated according to Olcott, et al.^49^.

Lifespan was calculated using the protocol of Linford, et al.^50^. For the calculation of adult survival rates, flies were monitored until natural death occurred. For analysis, the data was processed using the survival analysis tool of GraphPad Prism (version 10.2.2). In the software, Kaplan–Meier curves were calculated and analysed using the log-rank test with Mantel-Cox Test.

### Hypoxia experiments

On the fifth day after egg deposition, L3 larvae were exposed to hypoxia using a SICCO Mini 1 desiccator (Bohlender GmbH). 50 animals in vials with standard medium were positioned in the chamber and nitrogen was introduced until the oxygen level was reduced to 5%. Control animals were kept at normoxia (20–21% O_2_). Animals were kept at the respective oxygen conditions for two hours. During exposure, stress levels were measured by calculating the number of crawling larvae outside the medium as indicator for avoidance behavior.

### Statistics

All statistical analyses were performed with GraphPad Prism version 10.2.2. After testing for normality, Kruskal–Wallis test, one-way ANOVA and two-way ANOVA, followed by multiple comparisons test or log-rank test with Mantel-cox test were used where appropriate. Test details are described in the respective figure legend. For better readability, we have transformed data of some figures into percentages after statistical analysis. If so, this is indicated in the respective figure legends.

## Results

### Modified airway expression of spn43Aa or CG14933 alters the structure of larval airway branches

In a first step we quantified the mRNA expression levels of all candidate genes, including *spn43Aa* and *CG14933* in whole L3 larvae and isolated tracheae of wild-type Canton S flies (Fig. S1). Thereby we confirmed that the genes are expressed in the trachea. Next, we tested the overexpression and knockdown phenotype in the gene-modified fly lines. Expression of the gene in knockdown flies was statistically lower than in the genetic control, while expression of the genes in overexpression flies was statistically higher in comparison to the genetic control (Fig. S2).

Afterwards, we investigated if the modified expression of the respective genes each affect the airway structure. The larval respiratory system consists of two main tubes, that form the dorsal and lateral trunks and connect anterior openings in the thoracic segment to posterior openings in the abdominal segment. Primary tubes branch off both trunks and ramify into single cell secondary and tertiary branches. Blind-ending, finger-shaped terminal airway cells protrude in the tissue for oxygen supply.

**Figure 1:**
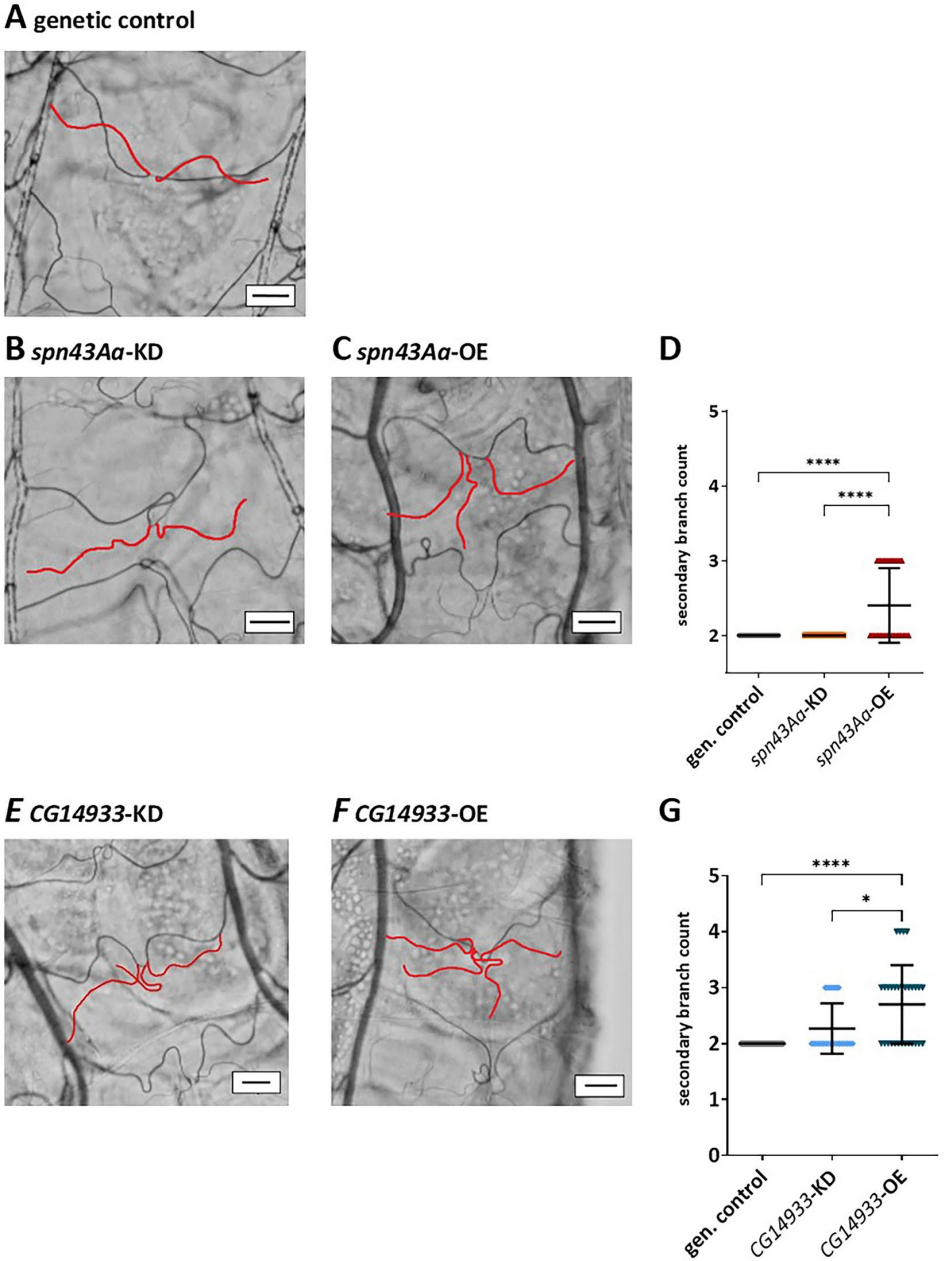
L1 Larvae with increased *spn43Aa* or *CG14933* expression develop additional secondary respiratory branches. Light microscopic images of secondary branches (marked in red) of the third thoracic segment in 1st instar larvae with altered *spn43Aa* (**B**,**C**) or *CG14933* (**D**,**E**) expression compared to genetic control animals (**A**). Scalebar shows a length of 20 µm. Statistical analysis showed a significantly increased numer of secondary branches in flies with *spn43Aa* overexpression (**D**), as well as in flies with overexpression of *CG14933* (**G**). n = 3 biological replicates (10 larvae per replicate). Shown is the mean ± SD; Statistical analysis was done with Kruskal–Wallis test followed by Dunn's multiple comparisons test. Asterisks indicate significant differences: ns > 0.05; *0.05–0.01; **0.01–0.001; ***0.001–0.0001; ****< 0.0001.

**Figure 2:**
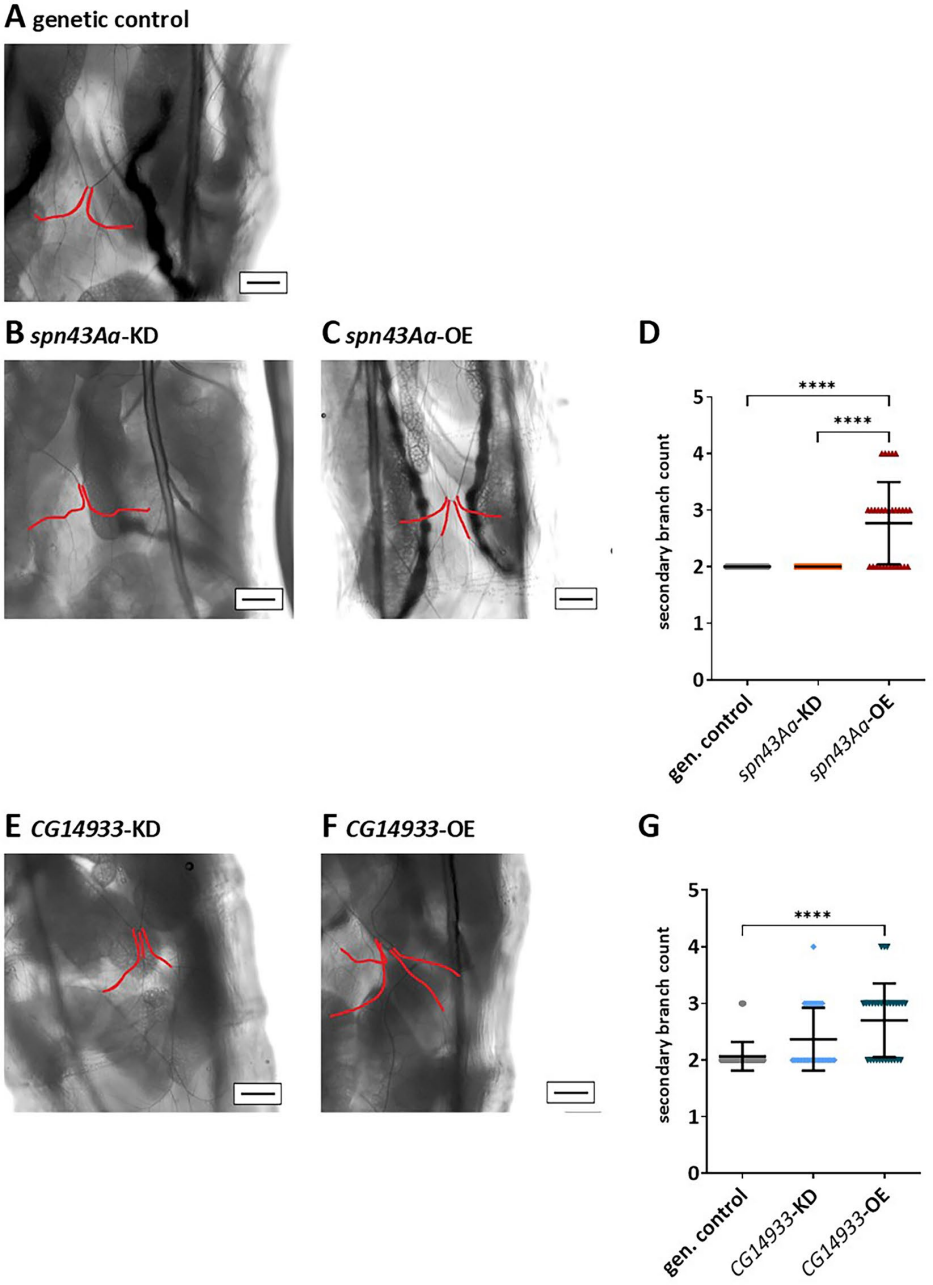
L3 Larvae with increased *spn43Aa* or *CG14933* expression develop even more additional secondary respiratory branches. Light microscopic images of secondary branches (marked in red) of the third thoracic segment in 3rd instar larvae with altered *spn43Aa* (**B**,**C**) or *CG14933* (**D**,**E**) expression compared to genetic control animals (**A**). Scalebar shows a length of 100 µm. Shown is the mean ± SD; Statistical analysis was done with Kruskal–Wallis test followed by Dunn's multiple comparisons test. Asterisks indicate significant differences: ns > 0.05; *0.05–0.01; **0.01–0.001; ***0.001–0.0001; ****< 0.0001.

Larvae overexpressing either *spn43Aa* or *CG14933* developed additional secondary branches (Figs. 1, 2). Thus, 40% of L1 larvae with *spn43Aa* OE had one extra secondary branch (Fig. 1C,D) which were additionally slightly increased in length (Fig. S3A). This morphological abnormality progressed with further development as 12.5% of L3 larvae had acquired two additional branches (Fig. 2C,D). Interestingly, the branches of *spn43Aa* OE larvae now became shorter, which was however not significant (Fig. S3C). In contrast, KD of *spn43Aa* did not affect the number of secondary branches in L1 (Fig. 1B,D) or L3 larvae (Fig. 2B,D).

Similar to *spn43Aa,* larvae overexpressing *CG14933* grew additional secondary branches in L1 (Fig. 1F,G ) and L3 larval stages (Fig. 2F,G). Thus, 43% of L1 larvae had one additional branch and 13% two extra branches. As for *spn43Aa* OE, more branches developed in *CG14933* L3 larvae, where 50% of larvae had three and 10% even four secondary branches (Fig. 2F), whereas only 6% of genetic controls had three branches. Different from *spn43Aa,* knockdown of *CG14933* also induced one additional branch in 23% of larvae in L1 stage (Fig. 1E), and one and two extra branches in 27% and 3% of L3 larvae, respectively (Fig. 2E) and reduced the length of secondary branches in 23% of L3 larvae (Fig. S3D).

### Changed expression of spn43Aa or CG14933 delays larval and pupal development and reduces their survival

**Figure 3:**
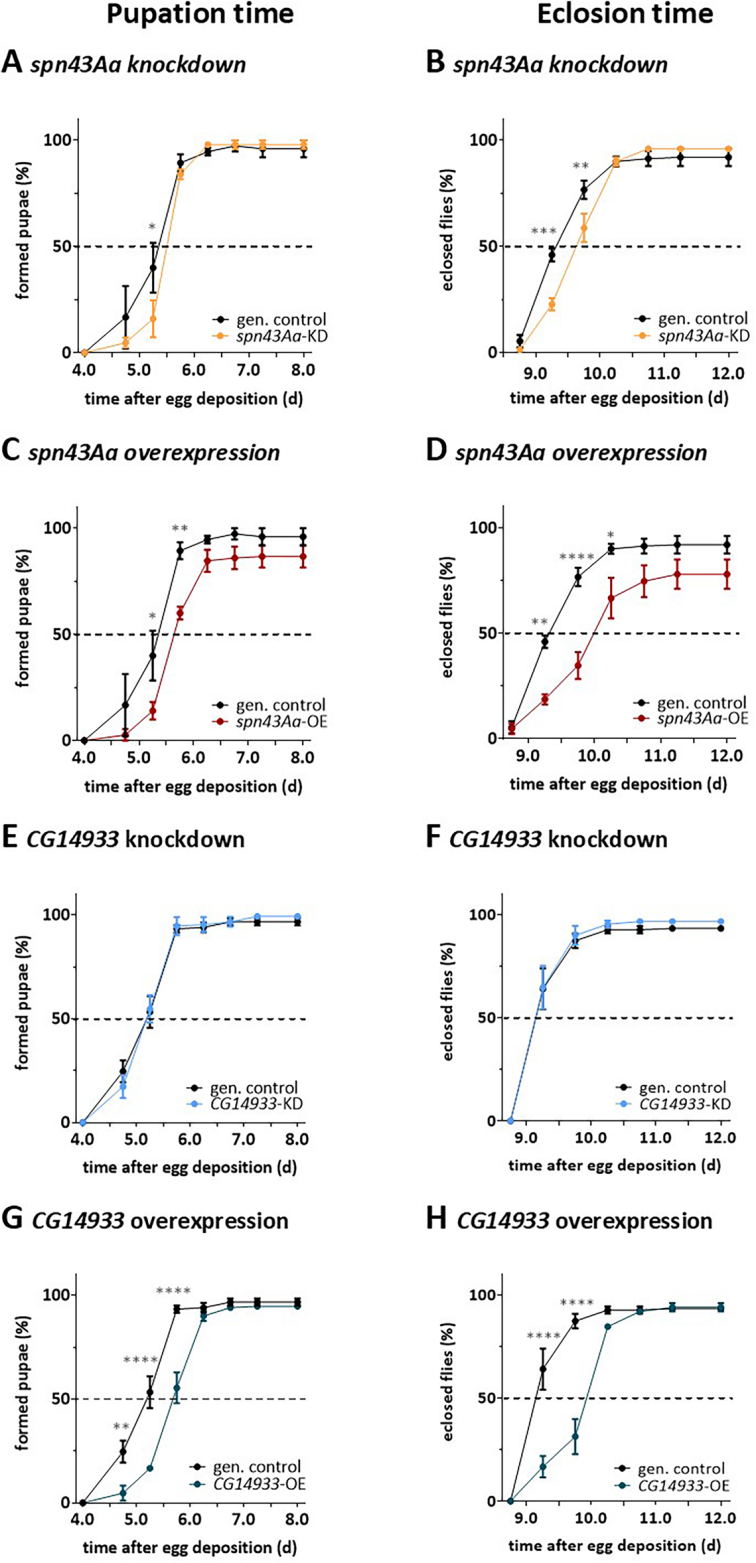
Larvae and pupae with modified expression of *spn43Aa* or *CG14933* in the respiratory tract require more time for development. Influence of modulated *CG14933* and *spn43Aa* expression in the respiratory tract on the developmental times of *Drosophila*. Shown are the percentages of pupae formed (**A**,**C**,**E**,**G**) and adults eclosed (**B**,**D**,**F**,**H**) at defined time points after egg deposition in fly lines with changes in *spn43Aa* (**A**–**D**) or *CG14933* (E–H) expression, each compared to the genetic control. n = 3 biological replicates (50 animals per replicate). Mean ± SD, original data was analysed with two-way ANOVA followed by Šídák's multiple comparisons post-hoc test. For purpose of better readability, data was transformed to % values afterwards. Asterisks indicate statistical significance: ns > 0.05; *0.05—0.01; **0.01—0.001; ***0.001—0.0001; ****< 0.0001.

To investigate if the observed altered airway morphology translates into reduced physiological fitness, we monitored the duration of the different developmental stages and of metamorphosis by recording the number of pupae and eclosed flies at specific timepoints after timed oviposition. Flies with dysregulated *spn43Aa* expression had a general delay in their development. While in genetic controls, 40% of the larvae went into pupation at day 5.25 after oviposition, only 16% and 14% of larvae with respectively reduced and increased expression of *spn43Aa* (Fig. 3A,C) were able to do so within this time. Their metamorphosis was prolonged as well upon dysregulation of *spn43Aa.* Thus*,* 46% of flies had eclosed after 9.25 days in genetic controls, which reached 77% half a day later. In contrast, only 23% of the flies with loss of *spn43Aa* had eclosed after 9.25 days reaching only 59% after 9.75 days (Fig. 3B). In flies with *spn43Aa* overexpression this effect was even more prominent as only 19% (day 9.25) and 35% (day 9.75) of the pupae were able to eclose (Fig. 3D). Overexpression of *CG14933* strongly delayed the transition of larvae into pupae and also of their metamorphosis (Fig. 3G,H) which was not seen after knockdown (Fig. 3E,F). We next asked if the observed developmental delays also result in reduced larval and pupal survival. The percentage of larvae dying before pupation was higher in animals overexpressing *spn43Aa* (median survival 86.4%) compared to genetic controls (median survival 97.2%) albeit not reaching statistical significance (Fig. S4A). Additionally, the percentage of dead pupae and consequently fewer adult flies was also increased in this genetic group (mean 84.8%), which however was not significant (Fig. S4B). This was not observed in flies with *spn43Aa* knockdown or dysregulated expression of *CG14933* (Fig. S4).

### Gene modification changes the lifespan of flies

**Figure 4:**
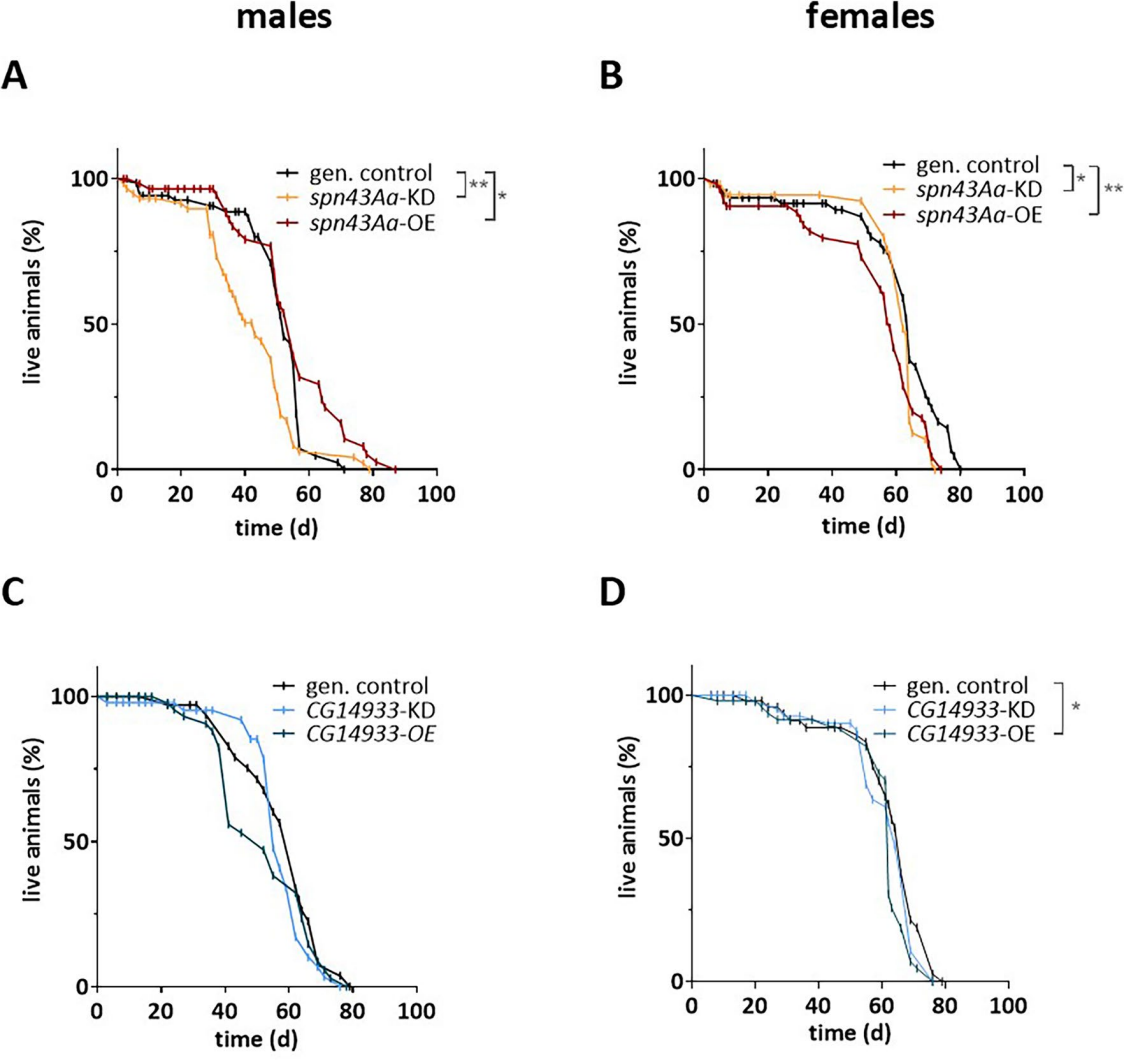
Flies with dysregulated expression of *spn43Aa* and overexpression of *CG14933* have a reduced lifespan. Survival of *Drosophila* adult flies with modified *spn43Aa* (**A**,**B**) or *CG14933* (**C**,**D**) expression in the respiratory tract. Shown is the percent of living males (**A**,**C**) and females (**B**,**D**) over time in. n = 3 biological replicates (20 animals per replicate). Kaplan–Meier curve, log-rank with Mantel-Cox test, ns > 0.05; *0.05–0.01; **0.01–0.001; ***0.001–0.0001; ****< 0.0001.

During pupal metamorphosis, airway progenitor cells, the tracheoblasts, give rise to the adult tracheal system by using parts of the larval system as scaffold^51^. Of note, the airway system of adult flies is much more complex than that of larvae^52^. Having shown that development is disturbed in gene-modified flies, we next monitored the lifespan of male and female adult flies until natural death occurred. A log-rank test was performed to assess whether significant differences exist between the gene-modified fly lines and the genetic controls. Results show that survival distributions differ significantly such that survival of males with knockdown of *spn43Aa* was significantly reduced to a median survival of 43 days, as compared to the median survival of 52 days in genetic controls. In contrast to that, median survival of males with overexpression of this gene was slightly prolonged to 55 days in comparison to the genetic control, which is most probably accountable to the extended terminal lifespan (Fig. 4A). In females, overexpression of *spn43Aa* significantly reduced median survival to 58 days, in comparison to the genetic control females with a median survival of 64 days. Likewise, for *spn43Aa* knockdown, median survival was slightly decreased to 62 days (Fig. 4B). For *CG14933* median survival was slightly reduced to 62 days in females overexpressing this serpin, whereas in males there was no statistical difference, even though median survival was reduced by 10 days (Fig. 4C,D).

### Dysregulation of spn43Aa and increased expression of CG14933 reduces larval tolerance to hypoxia

**Figure 5:**
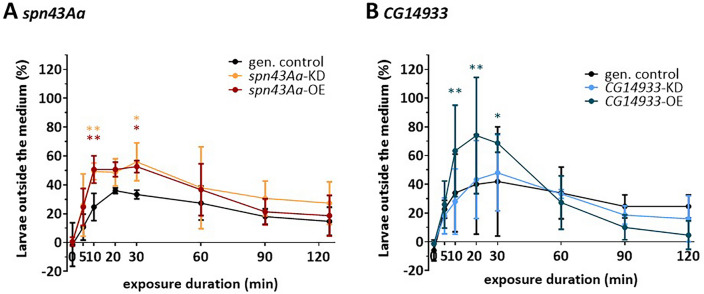
Influence of altered *spn43Aa* (**A**) or *CG14933* (**B**) expression on larval behavior under hypoxic conditions. Shown are the percent of larvae outside the medium (behavioral response) at defined time points during hypoxia exposure (5% O_2_, 2 h). Data was normalized to the respective normoxia group (ca. 21% O_2_). n = 3 biological replicates (50 animals per replicate). Shown are Mean ± SD. Statistical testing was done using two-way ANOVA, followed by Bonferroni's multiple comparisons test, asterisks indicate statistical significance ns > 0.05; * 0.05–0.01; ** 0.01–0.001; *** 0.001–0.0001; ****< 0.0001. For purpose of better readability, data was transformed to % values.

Having shown that dysregulation of *spn43Aa* and *CG14933* affects the airway structure as well as parameters of fitness and survival, we next aimed to elucidate if the latter is caused by a lower capacity for oxygenation. Under natural conditions, *Drosophila* larvae grow on rotting food with low ambient oxygen and have therefore evolved mechanisms to tolerate hypoxia^53–55^. As an indirect measure of tissue oxygenation, we exploited the phenomenon that larvae crawl to the surface of their medium under hypoxic conditions to achieve better oxygenation by gaining access to the air. In fact, when oxygen levels were artificially reduced to 5%, between 46 and 58% more larvae with either OE or KD of *spn43Aa* crawled out of the medium after 10 min (p ≤ 0.01) compared to genetic controls (Fig. 5A). After 30 min under hypoxic conditions, 44 to 70% of larvae with *spn43Aa* KD were found outside the medium (p ≤ 0.01), while 48 to 56% of *spn43Aa* OE larvae were likewise outside the medium (p ≤ 0.01) (Fig. 5A). Larvae overexpressing *CG14933* showed an equally strong behavioral response to the hypoxic condition. After 10 min of hypoxia, between 44 and 50% of larvae were found outside the medium (p ≤ 0.01) (Fig. 5B). 20 min after hypoxia started, between 44 and 60% of the larvae were outside the medium (p ≤ 0.01). The effect slightly decreased after 30 min, where between 38 and 66% of larvae were outside the medium (p ≤ 0.05) (Fig. 5B). In contrast to that, *CG14933* knockdown did not show any significant effect (Fig. 5B).

## Discussion

The present study shows for the first time that airway-specific deregulation of the two clade B serpins, *Spink5* and *Scca1*, alters the airway morphology in *Drosophila melanogaster* larvae, resulting in lower tolerance to hypoxia. We further observed delayed developmental stages and a reduced lifespan of adult flies. The strength of *D. melanogaster* is that it allows the airway-specific modification of genes involved in respiratory diseases along with direct visualization of the branching tree.

Larvae overexpressing the *Scca1* orthologue *spn43Aa* developed more secondary airway branches, that however were shorter in length. This demonstrates that a balanced expression of *spn43Aa* is crucial for optimal airway morphogenesis.

As in the human respiratory tract, the fruit fly’s airways develop from a series of epithelial branching events to finally form a functional respiratory tree. The molecular processes occurring during embryonic airway development of the fruit fly are highly defined in space and time. The expression of the *Scca1* orthologue *spn43Aa* in the airway epithelium is under the control of the transcription factor *trachealess* (trh) which is central to embryonic airway development^56,57^ as it regulates a multitude of genes involved in tracheal development. This includes the regulation of *breathless* (*btl*) which is a homologue to human Fibroblast Growth Factor Receptors (FGFRs). Among other molecules, btl/FGFR activates *pointed* and *sprouty*, both of which control the development of secondary branches, albeit in opposite directions^58^. Thus, *pointed* promotes the formation of secondary branches^59^, but also activates *sprouty*, which in turn inhibits *btl*, thereby creating a negative feedback loop that constrains the growth of secondary branches to the area closest to the *btl* signal^60^. Since overexpression of *spn43Aa* increased the numbers of secondary branches, *spn43Aa* could act as activator on *btl* signalling, and/or inhibitor of *sprouty* and thus interrupt the negative feedback loop. Increased length of secondary branches in L1 larvae with *spn43Aa* OE might be linked to the *btl* signalling pathway too, as *btl* controls the length of tracheal branches by promoting branch growth on the one hand and at the same time preventing an overshooting apical cell growth by activating its own antagonist *grainy head* (*grh)* on the other hand^61^.

Similar to *spn43Aa* OE, the dysregulated expression of the *Spink5* orthologue *CG14933* increased the number of secondary branches during L1 and L3 larval stages. Different from *spn43Aa* OE, however, branches in larvae with OE were normal in length while those of KD animals were shortened.

The development of additional branches might again be under the control of the above mentioned *btl*-signalling pathway, but the latter cannot explain the shortened branches seen in KD animals. The tube length is usually controlled by restricting the surface area of epithelial cells. Potential molecular players limiting the epithelial surface include the loss of the tyrosine kinase *Src42*^62^ or the evolutionarily conserved hippo-signalling pathway that is also involved in the regulation of the airway length^63^ and controls organ size in many organisms. Finally, the wingless (wg)/wnt-pathway is particularly active in the secondary branches^64^. In humans, *Spink5* has been shown to inhibit Wnt/β-catenin signalling pathway^65,66^ supporting the notion that the wg-pathway might be interconnected with *CG14933*. However, at present it is speculative if the *Spink5* homologue *CG14933* is directly involved in the regulation of the three pathways and will require deeper investigation in follow-up work.

We recognise that our study has some limitations that call for caution. This caution mainly concerns the choice of controls. Unfortunately, we used only some of the controls that we should have used in the optimal case, and therefore, there remains a degree of uncertainty regarding the observed phenotypes. However, our interpretation of the observed phenotypes is strengthened because we did not observe such changes in the parental lines.

The sufficient oxygenation of tissues is of vital importance for all living organisms that depend on gas exchange. As *Drosphilidae* feed on decaying food, they have evolved mechanisms to cope with very low ambient oxygen levels^67^. Larvae with any type of gene alteration had a greater need for oxygen, as reflected in their effort to crawl out of the medium under hypoxia.

This behaviour indicates that each of the two serpins is essential for the regular functioning of the respiratory tract and tissue oxygenation. So far, it is unknown if deregulation of *Spink5,* the human homologue of *CG14933,* also affects the airway function in humans, but gene variants for this gene have been reported for childhood asthma^30–33^.

The activity of the *ppk4-Gal4* is lost in major parts of the tracheal system of the adult fly, but remains in few epithelial cells^44^. Thus, the reduced lifespan might result from structural and functional impairments in those parts of the tracheal system that still express *ppk4*. The most marked morphological changes took place in the secondary branches. Since tracheoblasts are localised in proximity to secondary branches^51^, we speculate that molecular changes can be anchored in these cells that persist across metamorphosis and therefore could interfere with the formation of the adult airways and ultimately animal fitness. Additionally, as the larval tracheal system is at least partly used as scaffold for building the adult system, morphological changes in the larval system can potentially influence the structure of the adulty airway system.

In conclusion, our work contributes to the understanding of the functional role of *Spink5* and *Scca1* in the airway epithelium. The disturbed airway morphogenesis in our model evidently had a strong impact on the airway function as they manifested in a lower physical fitness of the animals with higher mortality of larvae and pupae as well as delayed development. In a translational aspect, the known dysregulation of *Spink5* and *Scca1* in children with asthma could therefore indicate to subtle deficits in the airway structure. In the future, the consequences of altered *Spink5* and *Scca1* need to be studied in disease models and under real-world exposures such as particulate matter or cigarette smoke.

### Supplementary Information

Supplementary Information.

## Data Availability

All data generated or analysed during this study are included in this published article [and its supplementary information files].
