# Supplementary material for: Airway specific deregulation of asthma-related serpins impairs tracheal architecture and oxygenation in D. melanogaster
Source: Sci Rep. 2024 Jul 17;14:16567. doi: 10.1038/s41598-024-66752-0 (PMC11255251; doi:10.1038/s41598-024-66752-0)
Supplement: Supplementary file 1 — Supplementary Information. [file 41598_2024_66752_MOESM1_ESM.pdf]

Supplemental Material

Airway specific deregulation of asthma-related serpins impairs tracheal architecture and oxygenation in *D. melanogaster*

Table S1: Overview of fly lines used in this study. Descripted is the genotype as well as the provider of the stocks. Additionally, information is given about genotypes of KD and OE lines.

Description	Genotype	Provided by
**GAL4-driver line** (for driving GAL4 in the tracheae)		
ppk4-GAL4	yw67c23;ppk4-gal4;+/+	Thomas Roeder; University of Kiel (Germany)
**UAS-RNAi lines** (for the UAS-dependent induction of gene knockdown)		
spn43Aa-RNAi	y[1] sc[*] v[1]; P{y[+t7.7] v[+t1.8]=TRiP.HMS02370}attP2	Bloomington Stock Center (Bloomington, US)
CG14933-RNAi	y1 v1; P{TRiP.HMJ23160}attP40	Bloomington Stock Center
Genetic control	y[1] v[1]; P{y[+t7.7]=CaryP}attP2	Bloomington Stock Center
**UAS-ORF lines** (for the UAS-dependent induction of gene overexpression)		
UAS-*CG14933*	M{UAS-CG14933.ORF.3xHA.GW}ZH-86Fb	FlyORF (Zürich, CH)
UAS-*spn43Aa*	M{UAS-Spn43Aa.ORF.3xHA.GW}ZH-86Fb	FlyORF (Zürich, CH)
**Genotypes after crossing**		
spn43Aa KD	*ppk4*-Gal4xUAS-*spn43Aa*-RNAi	
CG14933 KD	*ppk4*-Gal4xUAS-*CG14933*-RNAi	
spn43Aa OE	*ppk4*-Gal4xUAS-*spn43Aa*	
CG14933 OE	*ppk4*-Gal4xUAS-*CG14933*	

Table S2: qRT-PCR program used for quantification of gene expressionA

Step	Number of cycles	Temperature [°C]	Time [mm:ss]
**Denaturation**	1	95	10:00
**Amplification**	45	95	00:10
		63	00:10
		72	00:10
		78	00:01
**Melting curve**	1	95	00:10
		65	00:10
		95	00:10
**cooling**	1	40	∞

Table S3: qRT-PCR Primer. Primer were produced by Invitrogen Life Technologies (Karlsruhe). Sequences were searched with Flybase.org[1] and designed using the Primer design tool of the NCBI[2].

gene	Sequence (5' – 3')
Spn43Aa	sense: CCTTCATCGATGTGAACGAG
	antisense: GGAACTCCGGCAGCATAA
CG14933	sense: CATCCACACGTATAGAAAACATGAA
	antisense: AAAGAGGGCGATCAAAAGG
Rpl32	sense: CCAGTCGGATCGATATGCTAA
	antisense: GTTCGATCCGTAACCGATGT

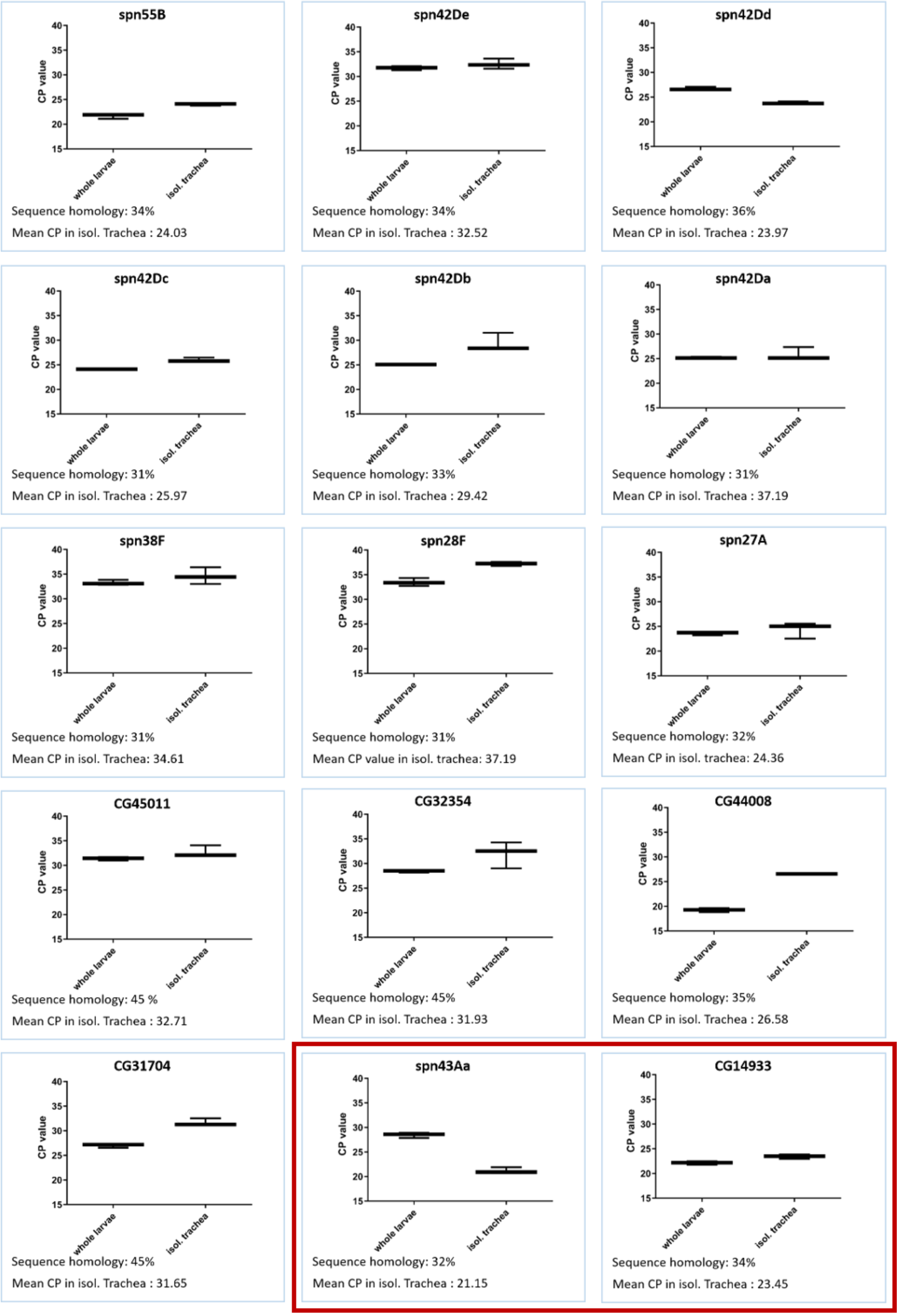

Figure S1: Expression levels of the candidate orthologs of *Scca1* (all spn genes) and *Spink5* (all CG genes) in whole larvae and isolated airways of *Drosophila melanogaster*. Candidate genes were selected based on the presence of an inhibitor activity and a sequence homology value > 30% (NCBI-BLAST). Shown is the mean CP value (n = 3, performed in triplicates) in whole

L3 larvae and isolated trachea of laboratory wild-type CantonS measured by qRT-PCR. Additionally, the percent sequence similarity value to human SERPINB3 and SPINK5 is shown. n = 3 biological replicates,each replicate presents 10 whole larvae or 40 dissected trachea. Median ± min/max. Based on the lowest CP value (=highest expression) in the tracheae, the two candidates *spn43Aa* and *CG14933* were chosen, which are are marked in red.

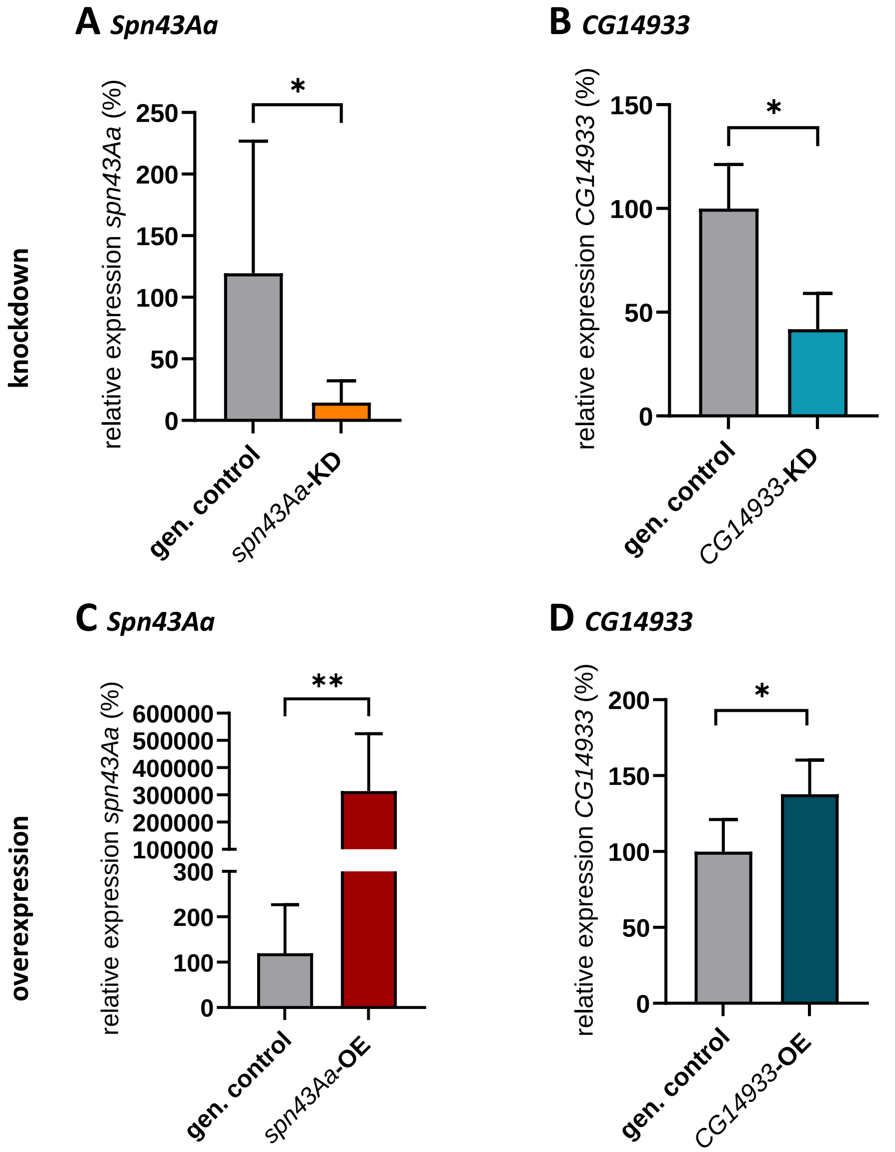

Figure S2: Relative expression levels after knockdown and overexpression of *Spn43Aa* (A, C) and *CG14933* (B, D). Expression was measured using qRT-PCR. Shown is expression of the target genes in isolated tracheae of genetic control and KD or OE flies, relative to the housekeeping gene *rpl32*. Data was calculated in %. Shown is mean ± SD. n = 3 biological replicates (40 isolated trachea per replicate), unpaired t-test was used for statistical testing, asterisks indicate level of significance: ns > 0.05; * 0.05 - 0.01; ** 0.01 - 0.001; *** 0.001 - 0.0001; **** <0.0001.

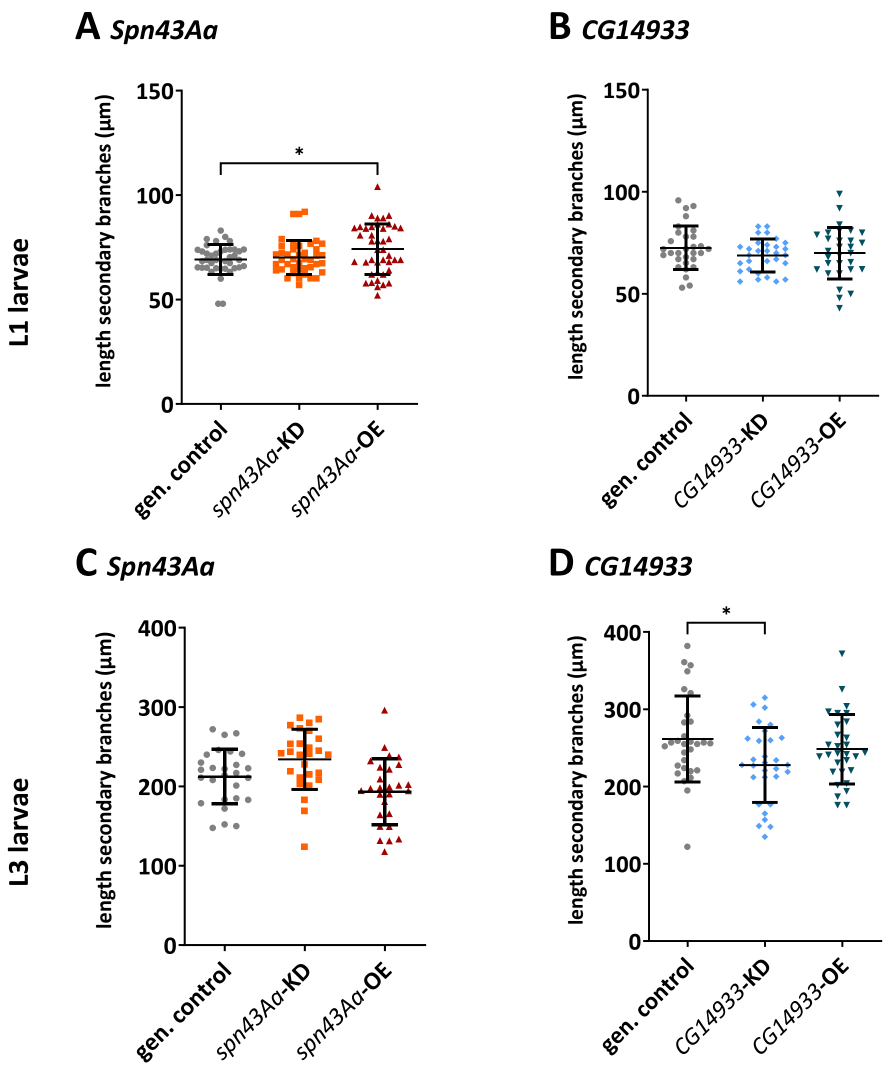

Figure S3: Length of secondary tracheal branches in 1st and 3rd instar larvae with modulated *spn43Aa* (A, C) or *CG14933* (B, D) expression. Shown is the length of secondary branches in L1 larvae of the genetic control, knockdown line (KD), and overexpression (OE) lines (A, B) and L3 larvae (C, D). n = 3 biological replicates (10 larvae per replicate). Shown is the Mean ± SD; Statistical analysis was done with one-way ANOVA, Dunnett's multiple comparisons test. Asterisks indicate significant differences: ns > 0.05; * 0.05 - 0.01; ** 0.01 - 0.001; *** 0.001 - 0.0001; **** <0.0001.

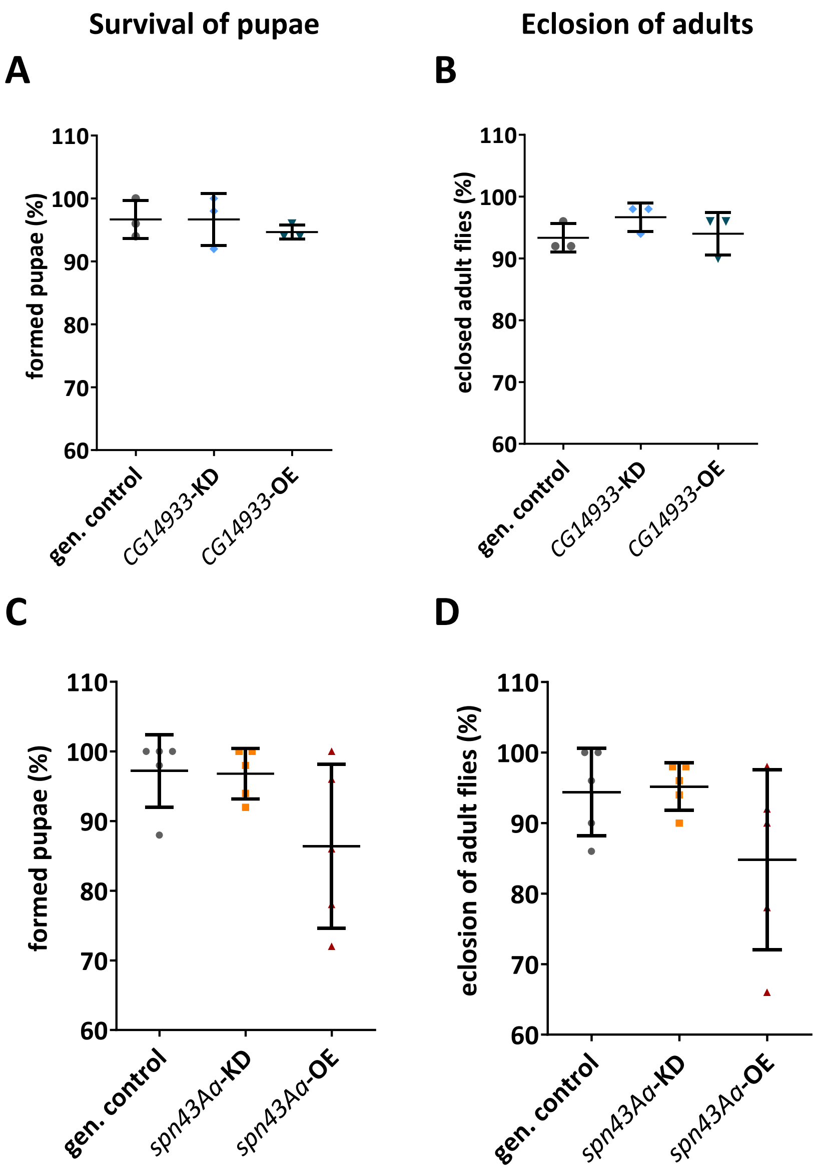

Figure S4: *Drosophila melanogater* with altered *CG14933* expression in the respiratory tract had no differences in pupation rate (A) or eclosion rate of adults (B). *Drosophila melanogater* with altered *spn43Aa* expression in the respiratory tract had a tendency for higher failure in pupation rate (C) or eclosion rate of adults (D). Shown is the percentage of living pupae (A, C) or adult flies (B, D) of the genetic control, knockdown (KD) and overexpression (OE) of *CG14933* and *spn43Aa*, relative to the initial number of animals. n = 3 biological replicates (50 animals per replicate). Mean ± SD. Data was analyzed with one-way ANOVA and transformed to percentages for better readability afterwards, ns > 0.05.

Supplemental literature

1 Gramates, L. S. *et al.* FlyBase: a guided tour of highlighted features. *Genetics* **220** (2022). https://doi.org/10.1093/genetics/iyac035

2 Ye, J. *et al.* Primer-BLAST: a tool to design target-specific primers for polymerase chain reaction. *BMC Bioinformatics* **13**, 134 (2012). https://doi.org/10.1186/1471-2105-13-134
